# The role of spirituality in improving psychosocial well-being in women with breast cancer: a qualitative study

**DOI:** 10.1007/s00520-026-10569-2

**Published:** 2026-03-17

**Authors:** Semra Seyhan-Şahin, Merve Işık

**Affiliations:** 1 Department of Nursing, Semra and Vefa Küçük Faculty of Health Sciences, Nevşehir Hacı Bektaş University, Nevşehir, Türkiye; 2 Department of Nursing, Faculty of Health Sciences, Giresun University, Giresun, Türkiye

**Keywords:** Spirituality, Breast cancer, Psychosocial well-being

## Abstract

**Purpose:**

Women with breast cancer face many psychosocial problems. This study aims to determine the spiritual experiences of women diagnosed with breast cancer and the effect of these experiences on their psychosocial well-being.

**Methods:**

The study was conducted with thematic analysis approach, and the sample consisted of women diagnosed with breast cancer (*n* = 15). The findings were obtained through individual semi-structured interviews, guided by the interview form presented in the Supplementary Materials. The thematic approach was employed to analyze data.

**Results:**

The main themes identified in this study were ‘spiritual empowerment and coping strategies during the breast cancer process’, ‘the impact of spirituality on social bonding and emotional support’, ‘empowerment and identity building through spirituality’, and ‘integration of spirituality into holistic cancer care’.

**Conclusion:**

Findings of the present study revealed that spirituality contributes to strengthening the emotional and social support mechanisms of patients, helping them develop coping strategies, and improving their psychosocial well-being. Raising the sensitivity of healthcare professionals to spiritual care and integrating it into patient care can help address the psychosocial needs of patients more comprehensively. Furthermore, promoting spiritual counselling services may improve the psychosocial well-being of patients.

**Supplementary Information:**

The online version contains supplementary material available at 10.1007/s00520-026-10569-2.

## Introduction

The diagnosis of breast cancer is generally recognized as a traumatic and life-changing experience for patients. Besides the main psychological symptoms such as anxiety, depression, anger, and worries about the future and death, patients may suffer from psychosocial problems such as impaired body image perception, reduced self-esteem and sexual difficulties [1]. The related studies have demonstrated that a great majority of patients with breast cancer struggle with a variety of psychosocial challenges such as anxiety, depression, stress, altered body image, fear of death, and social isolation [2]. In this sense, it has been increasingly stressed that the biomedical approaches alone are not sufficient and the psychosocial and spiritual needs of patients should also be taken into account and involved into support mechanisms [3].

Spirituality, particularly when coping with serious diseases, strengthens patients’ ties to life, enhances their hopes, and facilitates acceptance of the disease [3, 4]. Spirituality is a multidimensional construct covering meaning and purpose in life [5–7], connectedness to self, others, nature, or the sacred [8, 9], and transcendence beyond the self [10, 11]. It reflects an individual’s search for meaning, harmony, and connection beyond material existence. Religious beliefs or practices may express spirituality; however, spirituality also includes broader existential dimensions beyond religious coping [12, 13]. In this study’s cultural and clinical context, spirituality is conceptualized primarily through meaning and connectedness lens, with faith-based coping recognized as one of its behavioral manifestations. Spirituality is also an essential resource for alleviating distress and coping with the challenges of advanced-stage cancers [14–16] and has been associated with reduced negative emotions such as depression and anxiety, strengthened interpersonal relationships, and improved treatment adherence [14].

Several studies have demonstrated that spiritual experiences provide crucial psychological support for women with breast cancer in the early stages of the disease. For example, Devi and Fong [17] reported that Singaporean women with breast cancer reported profound spiritual needs within the first year after diagnosis, while Harrison et al., [18] noted that many of these needs remain unmet in similar populations. Nevertheless, spiritual dimensions are often underassessed or overlooked in oncology care [19, 20].

Spirituality may enhance psychosocial well-being through several interrelated pathways. Meaning-making and a renewed sense of purpose support emotional regulation and hope, helping patients to reinterpret suffering and sustain optimism throughout treatment [8, 21]. Connectedness—to God, to others, or existence—can reduce isolation and strengthen interpersonal bonds, thereby improving social functioning [9, 22]. Spiritual values such as acceptance, gratitude, and compassion further promote adaptive coping and engagement with care [5, 8]. Taken together, these processes form an integrative framework in which meaning-making and purpose foster emotion regulation and hope, perceived connectedness buffers isolation and strengthens relationships, and spirituality values foster adaptive coping and care engagement. Moreover, these mechanisms offer a conceptual framework for understanding how spirituality interacts with key domains of psychosocial well-being—emotional balance, social connectedness, and purposeful living. Involving these mechanisms into psychosocial care may therefore promote holistic recovery.

Existing literature on spirituality in breast cancer generally follows two distinct lines. The first line consists of studies that have investigated patients’ spiritual needs or spiritual distress, highlighting their frequent neglect in oncology settings [17–20]. The other comprises studies that have examined how spiritual meaning-making and lived spiritual experiences contribute to specific psychosocial domains such as emotional regulation and distress, relational functioning and support, and engagement with care and hope [23–26]. Specifically, qualitative insights remain limited on how these spiritual processes are experienced and operationally linked to these domains in breast cancer contexts. Accordingly, this qualitative study aims to explore how women with breast cancer interpret and embody spirituality as a psychosocial resource and how these experiences influence their emotional regulation, relational functioning, and engagement with care.

## Methods

### Aim of the study

This study aims to determine the spiritual experiences of women diagnosed with breast cancer and to examine how these experiences influence their psychosocial well-being.

### Study design

In this study, descriptive research design guided by a thematic analysis approach was used. Descriptive qualitative studies seek to explore participants’ thoughts, perceptions, and experiences regarding a particular phenomenon [27]. Accordingly, basic qualitative research design was preferred to determine the spirituality experiences of women diagnosed with breast cancer and the effect of these experiences on their psychosocial well-being. The study was carried out in accordance with the Consolidated Criteria for Reporting Qualitative Research (COREQ) checklist which allows reporting qualitative research in accordance with standards [28].

### Participants and setting

The study was conducted in the medical oncology unit of a university hospital in Turkiye. The study focused on determining the spiritual experiences of women diagnosed with breast cancer and their effect on psychosocial well-being. To ensure that participants could provide rich and various perspectives aligned with the research question, a mixed sampling approach (purposive–convenience) was adopted.

The sample consisted of women with a confirmed diagnosis of breast cancer who were receiving outpatient treatment in the medical oncology unit during the data collection. Participants were selected to capture variation in (a) diagnosis and disease stage (early vs. advanced), (b) treatment pathway (chemotherapy, radiotherapy, or hormone therapy), and (c) socio-demographic and experiential backgrounds (age, educational level, and individual spiritual experiences and meaning-making related to religious beliefs).

Although all participants were identified as Muslim, variation was observed in how religious beliefs were experienced, interpreted, and integrated into coping processes. Therefore, religious background was considered in experiential rather than denominational terms, which was aligned with the study’s focus on spiritual experiences rather than comparative religious analysis.

#### Recruitment framework and setting

Eligible participants were identified from the daily patient lists of the medical oncology unit by the assigned oncology nurses and the oncology secretariat, and their eligibility was verified by the research team. The recruitment process of the participants was conducted face-to-face in the outpatient clinic, where eligible patients were available.

#### Inclusion and exclusion criteria

Inclusion criteria were determined as follows: being 18 years of age or older, being diagnosed with breast cancer, being able to communicate in Turkish, and having sufficient cognitive capacity to participate in an interview. Patients with severe cognitive impairment, acute psychiatric symptoms, or medical instability that could prevent participation in the study were excluded.

#### Recruitment flow

During the recruitment process, a total of 22 women were invited to participate in the study. Eighteen women who met the inclusion criteria were assessed, and 15 of them granted their informed consent and participated in the study.

Data saturation was operationalized as a two-stage process. First, preliminary code saturation was defined as the point at which no new codes or concepts emerged across two consecutive interviews [29]. This approach is consistent with previous studies indicating that thematic and code saturation in qualitative studies is typically achieved within 12 to 20 interviews, depending on sample homogeneity and research scope [29, 30].

Although interviews were conducted by one researcher, data saturation was monitored by both researchers through concurrent data collection and analysis.After each interview, emerging codes were compared. Following the achievement of initial code saturation, recruitment continued to ensure confirmatory saturation, conceptual depth, and stability of themes. Recruitment was stopped when no new conceptual insights emerged and thematic redundancy was consistently observed across subsequent interviews.

A saturation monitoring table documenting the appearance and stabilization of new codes throughout interviews was used as a part of the audit trail and is presented in the Appendix 1 (Supplementary file).

All participants were informed about the study’s purpose, confidentiality was strictly maintained, and each participant was assigned a unique code (e.g., “P1”, “P2”, … “P15”).

### Data collection

Prior to the interviews, all participants were informed about the study’s purpose, and then they completed a personal information form containing questions about age, educational level, disease stage, and treatment.

Data were collected through in-depth individual interviews using a semi-structured interview guide. All face-to-face interviews were conducted in a quiet room within the oncology outpatient clinic to ensure participants’ comfort and confidentiality. After obtaining their verbal and written consent, each interview was audio-recorded using a digital voice recorder to ensure accurate data capture. Field notes were also taken to document nonverbal cues and contextual observations. The researchers started the interview with an icebreaker question to assess the impact of spirituality on participants’ psychosocial well-being during the disease process and encourage their involvement: “Would you like to share how your spirituality has affected your psychosocial well-being/treatment during your illness?” Appendix 2 presents the semi-structured interview form. (Supplementary file). However, some key sample questions are as follows: How did your support groups and the spiritual aspect of your family help you? How did your spiritual perspective support you against the changes you went through in your body during the disease process? The interviews lasted for approximately 35 min. The face-to-face interviews were held with the participants in the hospital environment where they could feel comfortable.

### Data analysis

The data were analyzed using the inductive thematic analysis method [31]. Following the interviews, the researchers transcribed the audio recordings verbatim. All interviews were conducted by a single researcher to ensure consistency and minimize interviewer-related bias. However, two researchers were involved in the data analysis process. Both of them read the transcripts line by line and independently coded the data using an inductive approach. After the initial coding, they compared and discussed the codes to identify similarities and differences. Discrepancies were resolved through iterative consensus meetings, during which code definitions were refined and merged when appropriate.

No formal intercoder reliability coefficient was calculated, as the analysis prioritized conceptual coherence and interpretive agreement rather than statistical precision. The final themes were reached by consensus and then organized under thematic categories supported by exemplar quotations from the participants’ narratives.

A detailed coding tree with exemplar quotations is presented in Supplementary file (appendix 3), and the data saturation monitoring table is included in Supplementary file (appendix 1).

### Trustworthiness

Credibility was supported through multiple strategies, including transcript verification and researcher triangulation. Interview transcripts were returned to participants solely to verify the accuracy of their statements, ensuring that their words were correctly transcribed. Member checking of codes and themes was not conducted due to the clinical workload and emotional sensitivity of the patient population, as additional follow-up participation could have imposed further burden on them.

To ensure the validity and reliability of the interview questions, the semi-structured interview form was reviewed by five experts from psychiatric nursing and qualitative research. Their feedback was used to refine the wording, sequence, and clarity of questions before data collection process began. This expert review process enhanced the content validity of the interview form and ensured that the questions were consistent with the study’s aims.

In the absence of member checking, credibility was strengthened through researcher triangulation, peer debriefing, and maintaining an audit trail. Two researchers independently analyzed the data, compared their interpretations, and discussed discrepant or negative cases until they reached a consensus. Dependability and confirmability were ensured through detailed documentation of analytic decisions and reflective memos. Transferability was promoted by providing rich contextual descriptions of the participants, setting, and sample characteristics.

### Ethical considerations

Approval was obtained from the Clinical Trials Ethics Committee of a university (Date: November 25, 2024/Decision No: 2024/14). Institutional permission was obtained from the related university hospital (Date: 23.12.2024/E-93596471–010.01–505705). All participants were informed about the purpose of the study and their personal information would be kept confidential. Written informed consent was obtained from all participants.

For ethical approval, the audio recordings of the interviews were securely stored on a password-protected computer accessible only to the research team. The recordings will be kept for five years following publication, and then deleted.

## Results

The sample consisted of 15 women with breast cancer, with a mean age of 46.40 years. Nine participants were high school graduates. Most of the participants (53%) were Stage II patients. Most of the participants were treated with chemotherapy and radiotherapy. All of them were Muslim (Table 1).

**Table 1:** Characteristics of the participants

ID code of the patients with breast cancer	Age	Educational level	Clinical stage	Treatment modality	Religion
P1	48	High school	Stage-II	RT + CT	Muslim
P2	55	High school	Stage-II	Surgery + RT + CT	Muslim
P3	52	High school	Stage -III	CT	Muslim
P4	44	University	Stage -III	CT	Muslim
P5	40	University	Stage -I	RT	Muslim
P6	56	High school	Stage-II	Surgery + RT + CT	Muslim
P7	50	Primary school	Stage -II	Surgery + RT + CT	Muslim
P8	42	High school	Stage -I	RT + CT	Muslim
P9	39	University	Stage -I	RT + CT	Muslim
P10	40	High school	Stage -II	Surgery + RT + CT	Muslim
P11	38	University	Stage -II	Surgery + RT + CT	Muslim
P12	43	High school	Stage -III	CT	Muslim
P13	56	High school	Stage -II	Surgery + RT + CT	Muslim
P14	52	University	Stage -II	Surgery + RT + CT	Muslim
P15	41	High school	Stage -I	RT + CT	Muslim

### Description of main themes

Four main themes came out of the data analysis: (1) spiritual empowerment and coping strategies during the breast cancer process, (2) the impact of spirituality on social bonding and emotional support, (3) empowerment and identity building through spirituality, and (4) integration of spirituality into holistic cancer care (Table 2).

**Table 2:** A summary of themes, sub-themes, and key codes

Themes	Sub-themes	Key codes
Spiritual empowerment and coping strategies during the breast cancer process	Psychological adjustment and emotional empowerment	The alleviating role of spirituality on psychological symptoms Achieving emotional well-being through faith Spirituality as a source of hope and strength Positive harmony and the search for meaning
	Spiritual awareness and the building of meaning for life	Strengthening of beliefs and existential questioning Increased spiritual awareness during the illness process Reevaluating the meaning of life from a spiritual perspective
The impact of spirituality on social bonding and emotional support	The role of spiritual and social support networks	Emotional support from family, friends groups Common spiritual values strengthens social bonding The role of support groups in psychosocial coping
	The healing effect of spiritual rituals	The calming and strengthening effects of prayer and worship Rituals supporting acceptance and inner peace
Empowerment and identity building through spirituality	Spiritual transformation of body perception and body image	The role of spirituality in coping with physical changes Transformation and acceptance in self-perception Gaining self-confidence and self-worth from a spiritual perspective
Integration of spirituality into holistic cancer care	The role of healthcare professionals in spiritual care	Understanding the spiritual needs of patients Care within the framework of spiritual values Health workers’ need for spiritual care competence
	Impact of spiritual support on the treatment process	Spiritual empowerment increases motivation and treatment adherence Faith-based informed decision-making process Spiritual support strengthens psychological resilience
	The supportive role of spiritual guidance during the patient care process	Spiritual counseling and guidance The healing effect of community-based spiritual guidance

#### Theme 1: spiritual empowerment and coping strategies during the breast cancer process

Women diagnosed with breast cancer reported that the diagnosis and treatment process were psychologically challenging. However, the use of spiritual and psychological resources during this process supported their psychological recovery.

### Psychological adjustment and emotional empowerment

The participants stated that they were able to control their feelings of anxiety, depression, and uncertainty—some of the psychological symptoms during the illness process— through spiritual beliefs and they were emotionally relieved. Spirituality was a source of hope and strength for the participants.

*“After my diagnosis, I was overwhelmed by fear, but embracing spirituality made me feel less alone and more able to cope.”* (P6).

Some participants stated that praying, worshipping, and holding on to their spiritual values provided them with tranquility and resilience.

*“I regained my hope by taking refuge in God. My illness wore me out psychologically but prayer kept me afloat.”* (P7).

### Spiritual awareness and the building of meaning for life

Some participants emphasized that their diseases caused them to question life and existence and led them to explore their spirituality more deeply.

*“Facing death pushed me further into my spirituality. I began to question my life and actually realized that every single moment was precious.”* (P2).

The participants also stated that by questioning the meaning of the challenging process they went through, they were able to create new meaning in their lives and their power to struggle with life improved.

*“I used to rebel against why this was happening to me. Over time, I held on to my spirituality and realized what I went through from a different perspective. My strength to hold on to life grew.”* (P6).

#### Theme 2: the impact of spirituality on social bonding and emotional support

Spiritual and social support networks were effective in coping with the disease. The emotional support from family and friend groups, together with the impact of spiritual rituals, strengthened the ability of participants to cope with the difficult process.

### The role of spiritual and social support networks

Family and friend groups that gave moral support to the participants contributed to their emotional empowerment during their illness.

*“The prayers and good wishes of my family and friends made me feel that I was not alone throughout this process.”* (P9).

Some participants emphasized that support groups that share common spiritual values were effective in psychosocial coping. One participant stated:

*“Praying together with people who struggled with the disease also helped me to get through the process more easily. I believe that sharing and uniting for a common purpose in prayer conveys a healing power.”* (P3).

### The healing effect of spiritual rituals

It was found that practices such as prayer and worship created a relaxing and empowering effect on the participants and helped them feel better spiritually.

*“Praying before sleep brings me relief and peace, and I believe an invisible force is supporting me through this process.”* (P6).

#### Theme 3: empowerment and identity building through spirituality

While breast cancer has brought about physical changes in many women, it also had a significant impact on their self-perception and identity as women. However, the spirituality became a significant support mechanism during the process of accepting these changes.

### Spiritual transformation of body perception and body image

Some women reported that taking a spiritual perspective on the changes that their bodies were undergoing helped them to accept this process more easily. Some participants stated that they experienced a boost in self-confidence toward their bodies from a spiritual perspective. The participant expressed:

*“Losing my hair initially upset me, but I later accepted it as part of a spiritual transformation, recognizing the temporary nature of physical appearance.”* (P1).

*“*I once felt ugly looking in the mirror, but through a spiritual lens, I now see a strong woman*”* (P10).

#### Theme 4: integration of spirituality into holistic cancer care

It was emphasized that healthcare services should not only be limited to pharmacological treatment, but also consider the spiritual needs of patients. Also, treatment should adopt a holistic approach that integrates and supports spirituality.

### The role of healthcare professionals in spiritual care

The patients stated that healthcare professionals who were spiritually sensitive to them helped them feel more comfortable during the treatment.

*“A few warm words from the nurses and their support for prayer make a meaningful difference in my treatment.”* (P7).

*“When my nurse considers my spiritual needs during my treatment, my hope for recovery grows even more.”* (P13).

On the other hand, some participants stated that healthcare professionals failed to provide adequate spiritual care, and this reflected on the treatment process.

*“Treating only my body makes me feel incomplete when my soul is neglected*” (P15).

### Impact of spiritual support on the treatment process

The participants stated that spiritual support promoted their treatment adherence and raised their motivation.

*“I perceive this process as a test due to my spiritual belief. I keep on struggling.”* (P6).

A few participants also stated that their beliefs were effective in making conscious decisions during the adjustment to the disease and strengthened their psychological resilience.

*“My faith guided me during this process and helped me hold on to life and feel stronger.”* (P9).

### The supportive role of spiritual guidance during the patient care process

Spiritual counselling and guidance, especially in the advanced stages of the disease, led patients to feel better psychosocially. Some participants expressed that these services should be expanded in order to control their fear of death.

*“The word ‘cancer’ means death for me. I find it difficult to manage sometimes. Spiritual guidance centers are needed”* (P11).

Some participants also highlighted the importance of meeting with other individuals, who shared similar experiences, to stay hopeful and to strongly overcome the disease process. They expressed that they needed referral from healthcare professionals for these services.

*“Connecting with people like me empowered me. We share the same hardships and rely on one another’s ideas and opinions.” (P2)*

*“I would like either nurses or doctors to introduce us to people who had similar illnesses and have been through this process.”* (P3).

## Discussion

The findings of this study suggested that spirituality played an important role in improving the psychosocial well-being of women with breast cancer. The participants stated that spiritual strength, coping strategies, and social support systems positively affected their psychological well-being during the disease process. These findings suggest that spirituality is a critical component that needs to be integrated in holistic care.

The findings of the study showed that the women diagnosed with breast cancer considered spirituality as a means of psychological and emotional empowerment. Spiritual belief alleviated anxiety, depression and social isolation and strengthened psychosocial well-being. As revealed by similar results in related studies, spirituality improves psychosocial well-being, boosts positive adjustment mechanisms in cancer patients, and promotes their development [32, 33]. The participants stated that while gaining spiritual awareness and building the meaning of life, they reconsidered the meaning of their lives with the reinforcement of their beliefs, and their spiritual awareness grew following the diagnosis. These findings suggest that adopting spiritual values is a significant coping mechanism for patients while learning to live with cancer.

Moral and social support from family and peer groups is a critical element for patients. Spiritual support groups bring hope and strength to patients, while social support networks improve their psychological resilience [34, 35]. Engaging in spiritual practices, including prayer and worship, can promote relaxation and healing, enabling patients to better cope with difficulties [36].

Moreover, the conscious decision-making process based on spiritual beliefs contributes to patients making decisions more consciously and safely. Spirituality helps them to be more resistant to challenging treatment processes by improving their psychological resilience [37].

The positive effects of spiritual counselling and peer support on the psychological well-being of cancer patients have been reported. Spiritual support is a critical factor in enhancing patients’ quality of life and managing the side effects of treatment. These findings suggest the need for greater integration of spiritual support services in health systems [38, 39].

The impact of spiritual belief on body perception and self-image was also a striking finding. Women with breast cancer who go through physical changes during their treatment accept these changes by attributing a spiritual meaning to them. This contributes to the improvement of self-worth and self-esteem and promotes the psychological healing. Spiritual belief allows individuals to reshape their identities and improves their psychological resilience [40].

The need for integrating healthcare professionals more into spiritual care process was another major issue emphasized by the participants. The understanding of the spiritual needs of patients and improving the spiritual care capabilities of healthcare professionals will contribute to the development of holistic patient care [41].

Consequently, it was observed that spirituality had a key role in improving the psychosocial well-being of women with breast cancer. Spirituality increases the adjustment of patients to the disease process in both individual and social dimensions and strengthens their psychological resilience. Involving spiritual care more in healthcare services and training healthcare professionals on this issue will contribute to improving the psychosocial well-being of patients.

### Limitations and strengths

The major strength of the study is that the role of spiritual support experiences of women with breast cancer on psychosocial well-being was acquired through qualitative interviews. Another strength is that this study indicated important findings on how healthcare professionals can use spiritual support approaches to improve the psychosocial well-being of patients. On the other hand, the fact that the study was conducted with breast cancer patients in a specific geographical region limits the applicability of its findings.

### Implication for nursing practices

Based on the findings of the study, spirituality is an important tool in improving psychosocial well-being. Efforts should be made to raise healthcare professionals’ sensitivity to spiritual care and to integrate spiritual care into healthcare services. Spiritual support groups and counselling services should be promoted to improve the psychosocial well-being of cancer patients. Individualized care plans that take patients’ spiritual beliefs into account should be designed. The role of spiritual guidance during treatment process should be increased, and policies should be developed accordingly.

## Conclusion

Findings of the present study indicated that spirituality was a powerful tool to promote psychosocial well-being, and patients expected healthcare professionals to integrate spiritual support into care during the treatment process. Furthermore, spirituality was an effective coping mechanism for patients, thus they reassessed their lives from a spiritual perspective. Accordingly, the importance of faith-based decision-making at every stage of treatment was highlighted. Accordingly, healthcare professionals caring for cancer patients should develop skills to improve their spiritual sensitivity. Furthermore, guiding patients to spiritual counselling and peer support groups can strengthen their psychosocial well-being by integrating spiritual support into care.

## Supplementary Information

Below is the link to the electronic supplementary material.

ESM 1 (DOCX 23.3 KB)

## Data Availability

The data supporting this study is available through the corresponding author upon reasonable request.
