# Supplementary material for: The role of spirituality in improving psychosocial well-being in women with breast cancer: a qualitative study
Source: Support Care Cancer. 2026 Mar 17;34(4):330. doi: 10.1007/s00520-026-10569-2 (PMC12992388; doi:10.1007/s00520-026-10569-2)
Supplement: Supplementary file 1 — (DOCX 23.3 KB) [file 520_2026_10569_MOESM1_ESM.docx]

**Appendix 1. Data Saturation Monitoring Table**

| **Participant Code** | | **New Codes Identified** | **Cumulative Codes** | **Evidence of Repetition** | **Researcher Notes** |
| --- | --- | --- | --- | --- | --- |
| P1 | 12 | | 12 | - | Initial interviews revealed major codes on emotional coping, prayer, and family support. |
| P2 | 5 | | 17 | **-** | Added new insights on hope, gratitude, and treatment motivation. |
| P3 | 3 | | 20 | Partial overlap | Reinforced themes of acceptance and self-worth; minor new code: “inner peace.” |
| P4 | 2 | | 22 | High overlap | Similar to previous; no major new concepts. |
| P5 | 1 | | 23 | High overlap | Emphasized meaning-making through suffering. |
| P6 | 1 | | 24 | High overlap | Confirmed previously identified themes. |
| P7 | 0 | | 24 | \| Full repetition \| \| --- \| | No new codes; similar to P3–P6 on faith-based acceptance. |
| P8 | 0 | | 24 | \| Full repetition \| \| --- \| | Reinforced earlier codes; no new codes identified. |
| P9 | 1 | | 25 | High overlap | Reiterated hope and social support; one minor contextual code (“gratitude to doctors”). |
| P10 | 0 | | 25 | \|  \| \| --- \|  \| Full repetition \| \| --- \| | Confirmed saturation across major categories. |
| P11 | 0 | | 25 | \|  \| \| --- \|  \| Full repetition \| \| --- \| | No new codes; same thematic structure. |
| P12 | 0 | | 25 | \|  \| \| --- \|  \| Full repetition \| \| --- \| | - |
| P13 | 0 | | 25 | \|  \| \| --- \|  \| Full repetition \| \| --- \| | - |
| P14 | 0 | | 25 | \|  \| \| --- \|  \| Full repetition \| \| --- \| | - |
| P15 | 0 | | 25 | \|  \| \| --- \|  \| Full repetition \| \| --- \| | Saturation confirmed after P13–P15; recruitment stopped. |

**Appendix 2. Semi-structured interview questions**

1. How did your spirituality affect your psychological resilience/psychosocial well-being after receiving a cancer diagnosis?
2. How did the spiritual aspect of support mechanisms in your social environment, such as your family, friends, or support groups, help you?
3. Have there been any changes in your beliefs or your perspective on the meaning of life during your illness?

a. How have you started to evaluate your life or your view of the future from a spiritual point of view after your encounter with breast cancer?

1. How did your spiritual perspective support you in the face of the changes you experienced in your body during the disease process?
2. What is the impact of receiving spiritual support (prayer, spiritual counseling, clergy, etc.) on your adherence to treatment or your decisions during the treatment process?

**Appendix 3. Coding Tree Structure**

| **Main Theme** | **Sub-theme** | **Example Codes** | **Illustrative Quotations** |
| --- | --- | --- | --- |
| Spiritual empowerment and coping | Psychological adjustment and emotional empowerment | Hope, prayer, inner peace, faith as strength, acceptance | “I regained my hope by taking refuge in God.” (P7) |
|  | Spiritual awareness and meaning-making | Questioning existence, life reevaluation, meaning through suffering | “Facing death pushed me further into my spirituality.” (P2) |
| Social bonding and emotional support | Spiritual and social support networks | Family prayers, shared faith, connection with others | “The prayers and good wishes of my family made me feel not alone.” (P9) |
|  | Healing effect of rituals | Calmness through prayer, collective worship | “When I pray, I feel tremendous relief and peace of mind.” (P6) |
| Empowerment and identity building | Spiritual transformation of body perception | Self-acceptance, self-worth, resilience | “When I lost my hair, I learned to see it as part of a spiritual transformation.” (P1) |
| Integration in holistic care | Role of healthcare professionals | Nurse empathy, spiritual sensitivity, prayer facilitation | “A few warm words from the nurses made a big difference in my treatment.” (P7) |
|  | \|  \| \| --- \|   Spiritual guidance and support | Counseling, community-based support, hope sharing | “Talking to people like me empowered me.” (P2) |
